# Medical artificial intelligence readiness scale for medical students (MAIRS-MS) – development, validity and reliability study

**DOI:** 10.1186/s12909-021-02546-6

**Published:** 2021-02-18

**Authors:** Ozan Karaca, S. Ayhan Çalışkan, Kadir Demir

**Affiliations:** 1 Department of Medical Education, Ege University Faculty of Medicine, İzmir, Turkey; 2 Department of Computer Education and Instructional Technology, Dokuz Eylül University Buca Faculty of Education, İzmir, Turkey

**Keywords:** Artificial intelligence, Medicine, Readiness, Medical students, Medical education, Scale development, Validity and reliability

## Abstract

**Background:**

It is unlikely that applications of artificial intelligence (AI) will completely replace physicians. However, it is very likely that AI applications will acquire many of their roles and generate new tasks in medical care. To be ready for new roles and tasks, medical students and physicians will need to understand the fundamentals of AI and data science, mathematical concepts, and related ethical and medico-legal issues in addition with the standard medical principles. Nevertheless, there is no valid and reliable instrument available in the literature to measure medical AI readiness. In this study, we have described the development of a valid and reliable psychometric measurement tool for the assessment of the perceived readiness of medical students on AI technologies and its applications in medicine.

**Methods:**

To define medical students’ required competencies on AI, a diverse set of experts’ opinions were obtained by a qualitative method and were used as a theoretical framework, while creating the item pool of the scale. Exploratory Factor Analysis (EFA) and Confirmatory Factor Analysis (CFA) were applied.

**Results:**

A total of 568 medical students during the EFA phase and 329 medical students during the CFA phase, enrolled in two different public universities in Turkey participated in this study. The initial 27-items finalized with a 22-items scale in a four-factor structure (cognition, ability, vision, and ethics), which explains 50.9% cumulative variance that resulted from the EFA. Cronbach’s alpha reliability coefficient was 0.87. CFA indicated appropriate fit of the four-factor model (*χ*^*2*^*/df* = 3.81, RMSEA = 0.094, SRMR = 0.057, CFI = 0.938, and NNFI (TLI) = 0.928). These values showed that the four-factor model has construct validity.

**Conclusions:**

The newly developed Medical Artificial Intelligence Readiness Scale for Medical Students (MAIRS-MS) was found to be valid and reliable tool for evaluation and monitoring of perceived readiness levels of medical students on AI technologies and applications.

Medical schools may follow ‘a physician training perspective that is compatible with AI in medicine’ to their curricula by using MAIRS-MS. This scale could be benefitted by medical and health science education institutions as a valuable curriculum development tool with its learner needs assessment and participants’ end-course perceived readiness opportunities.

**Supplementary Information:**

The online version contains supplementary material available at 10.1186/s12909-021-02546-6.

## Background

Information processing technologies those were used to assist humanity in numerical calculations, have become instantaneously processing data that is too complex for the human brain to be calculated in parallel with the geometric increase in their capacities. In addition to banking, manufacturing, agriculture, transportation, education, psychology etc., artificial intelligence (AI) has started to influence the healthcare field over decades. Studies on AI in the field of medicine have a wide range from collecting daily health data, interpreting the data, and imaging (e.g., radiology and pathology), using them as supportive information in therapeutic and surgical procedures, and warning the patient and related personnel, when necessary. Since, one of the main application of AI is medicine and health sciences, it is an inevitable necessity for vocational education in this field to adapt to the developments related to AI [1].

Technology offers new solutions to improve healthcare quality and facilitates its access. Computer-assisted systems had been producing similar outputs to the human brain in healthcare services since the early 1970s [2]. Nowadays, many health conditions such as eye diseases, pneumonia, breast and skin cancers can accurately be detected by rapidly analyzing medical imaging with AI applications [3–7].. In addition, AI applications can detect coronary heart diseases by analyzing echocardiograms [8], psychotic events and neurological diseases such as Parkinson’s from speech patterns [9], facilitate the diagnosis of polyps and neoplasms in the gastrointestinal system [10] and perform certain procedural tasks such as knot tying during robotic surgery [11]. Also, AI has the potential to aid in early detection of infectious disease outbreaks [12] and its sources such as water contamination to protect public health [13]. Such AI applications might play an important role in reducing the patient care burden on many healthcare professionals or pave the way for reaching more patients [3].

In this context, employing AI in healthcare applications has generated great interest in last few years [14], and it can be conjectured that these AI-based healthcare/medical applications can help medical professionals to diagnose more reliably, improve treatment results, reduce malpractice risks and treat more patients [15]. Keeping current healthcare condition and advancements in view, it can be assumed that almost every type of clinician will be using AI technology for various purposes in the near future [3]. Considering all these assumptions, medical education - that is confronting with the potential and challenges of emerging technologies in AI- itself will be at the center of the seek for a solution [16]. Educational research is also needed to figure out, how AI will better impact medical education? [17]. Learning the fundamentals of AI will help students in grasping the effects of AI in relation to the daily medical procedures. However, medical students should be taught that promises of AI are limited and medical procedures are not simply statistical and procedural [2]. For instance, it is suggested that medical students should have prior knowledge of clinical AI systems and statistical modelling methods to test innovative AI technologies [18].

### Medical artificial intelligence readiness

Merriam-Webster dictionary defines *readiness* as “the quality or state of being ready”. In the educational context, readiness is considered an indispensable component of teaching and learning process [19]. The emergence of a new behavior change in the education depends on the student’s level of readiness. For this reason, a student must have cognitive, affective, and psychomotor behaviors, which is necessary for the acquisition of new behavior [20]. Since, education is a behavioral change process, so measuring readiness at the beginning of the process will help in identifying from where to start the training [21]. Measuring the level of readiness allows, beginning from the first day to provide guidance in accordance with the individual and characteristic features of the individual, to examine the needs of the individual and to make plans, programs, and preparations in accordance with these needs. Keeping aforementioned facts in view, describing the readiness of medical artificial intelligence will be a guide to work on this issue.

We propose **medical artificial intelligence readiness** is the healthcare provider’s preparedness state in knowledge, skills, and attitude to utilize healthcare-AI applications during delivering prevention, diagnosis, treatment, and rehabilitation services in amalgam with own professional knowledge.

Considering global AI boom in view, it is expected that AI will be the one of the main elements of medical education in the coming years [22]. Future physicians are supposed to be able to objectively analyze the use of AI systems, consider the discrepancies between algorithms generated for medical tasks, better understand AI, and thereby become educated users [23]. So, the measurement of perceived medical artificial intelligence readiness of medical school students is important to guide for various educational design and developmental processes such as curriculum development, instructional design or needs analysis, etc. Although, some researchers have tried to put forth the concurrent AI knowledge and attitudes of medical students [14, 24, 25]. However, to the best of our knowledge, there is no published medical artificial intelligence readiness scale available. In the present article we describe the development of a reliable scale for measuring the perceived medical artificial intelligence readiness of medical students and tested its validity.

## Method

### Research design

**Fig. 1:**
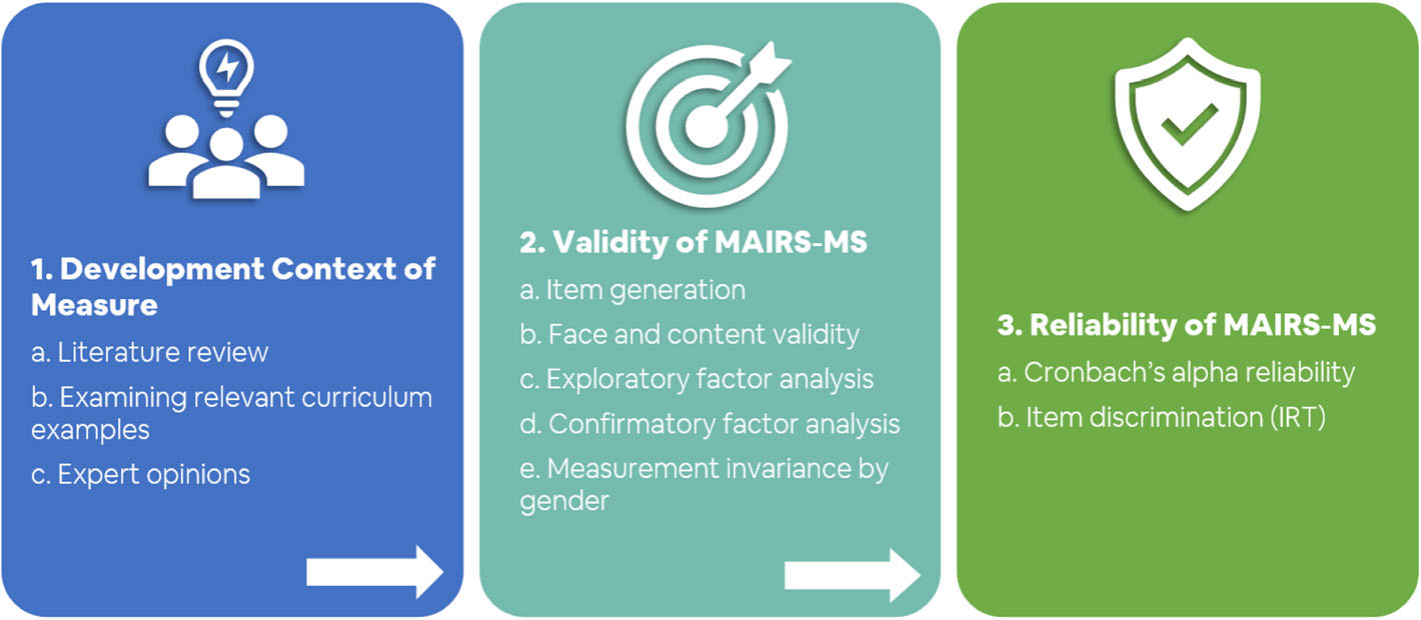
Phases and steps involved in perceived medical AI readiness scale development and its validation

This study was dedicated for the development of psychometric scale, followed by its validity and reliability studies using sequential exploratory mixed method [26]. According to the method, the main construct to be measured in this research was determined as perceived medical artificial intelligence readiness of medical students. An item pool was generated by an extensive literature search and expert opinions. Item format was determined following the Likert scale, using response options showing various levels of item engagement, and is frequently preferred in similar studies. The generated items were reviewed by the field experts and the initial scale was developed. The developed scale was evaluated for its validity and reliability employing Exploratory Factor Analysis (EFA) and Confirmatory Factor Analysis (CFA) and the final version was established as a reliable medical AI readiness scale (Fig. 1) [27].

### Participants

Medical students are considered to be appropriate sample as they are relatively homogeneous group according to the Turkey’s central university admission examination and student selection criteria. The data were collected from undergraduate medical students enrolled in two public universities in Turkey. The study was carried out with 568 participants of Ege University (EU) during the exploratory factor analysis (EFA) phase and with 329 participants of Manisa Celal Bayar University (MCBU) during the confirmatory factor analysis (CFA) phase. Medical students were accessed through convenience sampling via students’ classmate WhatsApp communication groups.

### Data collection and analysis

Both EFA and CFA were performed in June 2020 at EU and MCBU, respectively. A Microsoft Forms based online survey questionnaire (prepared in Turkish language) was sent to all the participants via WhatsApp communication groups. In addition to the demographic information, the participants were asked to rate all the items using a Likert-type rating scale (1-strongly disagree to 5-strongly agree). The participants sent all responses by entering the electronic survey form.

The quantitative data were analyzed using descriptive statistics. The factor structure of Medical Artificial Intelligence Readiness Scale for Medical Students (MAIRS-MS) was evaluated by principal component analysis followed by varimax rotation, by which the scale’s structural validity was assessed. MAIRS-MS factors were selected according to eigenvalues greater than 1.0, scree-plot test and with a value of Kaiser-Meyer-Olkin of 0.6 and above. The internal consistency of MAIRS-MS was evaluated by Cronbach’s alpha. The statistical analysis was performed by using IBM SPSS Statistics v21, Mplus 8.5 and R-4.0.3. The confidence interval (CI) was accepted as 99%, and *p* < 0.01 was considered statistically significant.

## Results

A total of 568 and 329 responses were received in EFA and CFA, respectively; out of which, 544 and 321 responses were valid (24 and 8 for EFA & CFA, respectively, were excluded due to missed values). The demographic characteristics of the participants included in the factor analysis have been summarized in Table 1.

**Table 1:** Demographic characteristics of participants (n_EFA_ = 544, n_CFA_ = 321)

Characteristics	EFA		CFA	
	N	%	N	%
Gender				
Female	274	50.4	172	53.6
Male	269	49.4	147	45.8
Unanswered	1	0.2	2	0.6
Age	20.95 (SD 1.99) years		21.94 (SD 1.82) years	
Medical School Year				
1st	111	20.4	42	13.1
2nd	129	23.7	45	14.0
3rd	100	18.4	19	5.9
4th	128	23.5	140	43.6
5th	51	9.4	58	18.1
6th	25	4.6	17	5.3

### Validity

#### Item generation

We sought the opinion of a diverse set of experts involved either using or developing AI in healthcare: (a) healthcare professionals/ academics; (b) computer and data science professionals/ academics; (c) law and ethics professionals/ academics; and (d) medical students. A purposeful snowball sampling method was used to identify and complete the expert group. A total of 94 participants comprised the expert panel. An online survey questionnaire was sent via email. In addition to demographic information, all the participants were asked to list all competencies that will enable medical students to be ready for artificial intelligence technologies and possible applications in medicine. Seventy-five (79.8%) expert panel members submitted a total of 220 phrases or sentences. These inputs were reviewed and revised by the researchers in terms of content and wording. The items covering the same or similar content were combined and a list of 41 initial items was obtained.

This initial item list was sent to two experts involved in using and developing AI tools/techniques in healthcare (one medical academic professional and one computer and data science academic professional) and their qualitative opinions were requested. Through the review, combining items, omitting items, and wording changes were suggested. These suggestions were incorporated into the list and a scale with 27 items was obtained.

#### Face and content validity

The newly developed AI readiness scale was then sent to seven experts (i.e., four field experts, two psychometricians, and a Turkish language specialist), who evaluated it for the content and wording. Thus, a peer evaluation provided with a critique of the items, instructions, and appearance of this new instrument was done. The qualitative evaluations proposed by the experts via an opinion form were examined by the researchers.

After verification by the expert panel, two medical students were requested to respond to the instrument followed by an interview in terms of gathering their reviews on semantic contents, clarity, and readability of the items. Some minor wording changes were applied according to their suggestions. The scale was then accepted to have adequate face and content validity with 27 retained items for EFA.

#### Exploratory factor analysis (EFA)

To evaluate the factor structure of 27 items’ scale, we performed EFA using varimax rotation with Kaiser-Meyer-Olkin normalization. Kaiser measure of sampling adequacy for EFA was 0.89 and Bartlett test of sphericity was significant, *χ*^*2*^_*(df = 231)*_ = 3711,19, *p* < 0.001. In order to consider different viewpoints for judicious preservation and deletion of the item, the analysis was done by the research team together. While performing the factoring process, eigenvalues were examined first. In addition to this process, Kaiser criterion [28] and the scree plot [29] were employed. Following these steps, the research team revealed that the scale was composed of a four-factor structure.

Exploratory factor analysis showed that five items were either loaded on more than a single factor and the loading difference was smaller than 0.10 or failed to load on any single factor (loading< 0.40) (Additional file 1). Four-factor structure explains 50.9% cumulative variance, which was resulted from the EFA phase after omitting such five items. The four-factor structure named cognition, ability, vision, and ethics constituted 16.60, 14.69, 10.65 and 9.05% of the explained variance, respectively. All communalities were higher than 0.26. The rotated factor matrix is presented in Table 2.

**Table 2:** Rotated factor matrix

Factors	1	2	3	4
***Cognition***				
I can define the basic concepts of data science.	**0.783**	0.073	0.153	−0.044
I can define the basic concepts of statistics.	**0.749**	−0.089	0.089	0.110
I can explain how AI systems are trained.	**0.679**	0.158	0.166	−0.006
I can define the basic concepts and terminology of AI.	**0.578**	0.294	0.236	−0.080
I can properly analyze the data obtained by AI in healthcare.	**0.555**	0.332	0.178	0.151
I can differentiate between the functions and features of AI related tools and applications.	**0.531**	0.383	0.332	−0.096
I can organize workflows in accordance with the logic of AI.	**0.457**	0.369	−0.037	0.256
I can express the importance of data collection, analysis, evaluation and safety; for the development of AI in healthcare.	**0.419**	0.353	0.310	0.006
***Ability***				
I can use AI-based information in combination with my professional knowledge.	0.176	**0.684**	0.117	0.024
I can use AI technologies effectively and efficiently in healthcare delivery.	0.278	**0.623**	0.239	0.267
I can use artificial intelligence applications in accordance with its purpose.	0.231	**0.563**	0.264	0.256
I can access, evaluate, use, share and create new knowledge using information and communication technologies.	0.152	**0.530**	0.003	0.087
I can explain how AI applications in healthcare offer a solution to which problem.	0.341	**0.530**	0.285	−0.096
I find it valuable to use AI for education, service and research purposes.	−0.014	**0.526**	0.010	0.241
I can explain the AI applications used in healthcare services to the patient.	−0.053	**0.510**	0.007	−0.060
I can choose the proper AI application for the problem encountered in healthcare.	0.361	**0.486**	0.189	0.087
***Vision***				
I can explain the limitations of AI technology.	0.250	0.087	**0.767**	0.081
I can explain the strengths and weaknesses of AI technology.	0.252	0.172	**0.758**	0.005
I can foresee the opportunities and threats that AI technology can create.	0.078	0.078	**0.692**	0.165
***Ethics***				
I can use health data in accordance with legal and ethical norms.	−0.025	0.138	0.202	**0.814**
I can act in accordance with ethical principles while using AI technologies.	−0.113	0.127	0.153	**0.811**
I can follow the legal regulations regarding the use of AI technologies in healthcare.	0.360	0.054	−0.162	**0.543**

Bold: Highest factor load

The frequencies of all the responses were reviewed for outliers and non-normality. The responses of EFA scale revealed acceptable skewness (0.040) and kurtosis (− 0.172) values, which meant that the means of the scale were normally distributed [30]. Tests of normality suggested that kurtosis and skewness coefficients ranged within the threshold values of ±3, and therefore, it can be said that the data was normally distributed.

#### Confirmatory factor analysis (CFA)

Before the CFA, the data were analyzed and the responses of the CFA scale revealed acceptable skewness (− 0.705) and kurtosis (1.228) values, that confirm the means of the scale were normally distributed [24]. In CFA, the data were analyzed using Mplus software. Since, the data were of ordinal in nature, weighted least square mean and variance adjusted (WLSMV) were used as the estimation method. Later, in order to further improve model fit, three associated error sources with statistical and contextual positive correlations were added to the model with the help of modification indices (Additional file 2). Since, the structural validity was obtained in the only model tested, new data were not collected.

When the fit indices of the model tested with CFA, it was found that the Chi-square value (*χ*^*2*^_*(df = 200)*_ = 762.203, *p* < 0.001) was significant. It was found that the calculated model *χ*^*2*^/*df* = 3.811 ratio indicated a perfect fit. It was observed that other model fitness values (RMSEA = 0.094, SRMR = 0.057, CFI = 0.938, NNFI = 0.928,) were all within the acceptable fitness interval as summarized in Table 3.

**Table 3:** Measures of model fit for the CFA model

Fit indicators	Observed Value	Acceptable value	Source
*χ*^*2*^*/df*	3.811	Between 1 and 5	[31] [32]
RMSEA	0.094	< 0.10	[33]
SRMR	0.057	< 0.08	[34]
CFI	0.938	> 0.90	[33]
NNFI	0.928	> 0.90	[33]

Note. *CFA* Confirmatory factor analysis; *x*^*2*^*/df* Residual degrees of freedom; *RMSEA* Root mean square error of approximation; *SRMR* Standardized root mean square residual; *CFI* Comparative fit index; *NNFI* Non-standard fit index

#### Measurement invariance by gender

The CFA model (Additional file 2) was tested for gender invariance. We followed the guidelines by Millsap and Yun-Tein [35] and completed the analyses using the semTools package [36] with WLSMV and the Satorra and Bentler [37] chi-square difference test. Strict invariance (*Δχ*^*2*^ = 26.59, *p* = 0.22) was evident, which indicates that gender-based differences in the total scores are not caused by a defect in the scale (Table 4) [38].

**Table 4:** MAIRS-MS Measurement invariance by gender

	*df*	*χ*^*2*^	*Δχ*^*2*^	*Δdf*	*p*
Structural	400	227.88			
Weak	418	277.53	16.88	18	0.53
Strong	436	284.98	15.26	18	0.64
Strict	458	300.49	26.59	22	0.22

### Reliability

#### Cronbach’s alpha reliability

The internal consistency coefficients of all the factors were found acceptable [27, 39]. The Cronbach’s alpha coefficient for the whole scale was 0.877. The Cronbach’s alpha coefficients were 0.830, 0.770, 0.723, 0.632 for cognition, ability, vision and ethics factors, respectively. The item loadings ranged between 0.419 to 0.814. Reliabilities of factors ranged from 0.632 to 0.830 (Table 5). Further, correlations between the factors were significant (*p* < 0.01). The factors were related with each other as summarized in Table 5.

**Table 5:** Descriptive statistics and reliability of factors

Factors	1	2	3	Mean	S.D.	Cronbach Alpha	Skewness	Kurtosis
1. Cognition				34.351	5.803	0.830	0.042	−0.099
2. Ability	0.677*			31.680	3.473	0.770	−0.108	0.139
3. Vision	0.490*	0.442*		10.831	2.050	0.723	−0.349	0.110
4. Ethics	0.208*	0.305*	0.209*	11.448	1.805	0.632	−0.627	1.758

*p < 0.01

#### Item discrimination

For the item discrimination values, a multidimensional item response theory (mirt) package [40] was used to estimate the graded response model separately for each dimension with the help of R software program. The item discrimination values were found consistent with the high item loadings in CFA with an average of 2.41 (Table 6).

**Table 6:** Scale item discrimination parameters

Item #	Discrimination parameter
1	1.44
2	1.46
3	1.72
4	2.15
5	2.39
6	2.83
7	2.33
8	1.85
9	2.95
10	3.23
11	4.10
12	2.04
13	1.90
14	1.43
15	1.65
16	2.10
17	2.33
18	4.05
19	2.50
20	2.89
21	4.33
22	1.38

## Discussion

Artificial intelligence is leading towards a new era that will reshape the medicine and healthcare delivery services in the coming years [2].. Although, it is not anticipated that AI will replace the role of physicians, but it will definitely undertake many tasks belonging to physicians, bringing healthcare services to a better level with faster pace, thus there is a need to create new tasks and new learning requirements for physicians, which will assist in reshaping their professional identities [2, 23, 41]. Learning the basics logic and pros/cons of machine learning, its applications at medical faculty will prepare future physicians for the data science revolution and AI competencies such as making data-based decisions [42–44]. Also in this way, students and medical professionals can acquire adequate skills to participate in the upcoming AI ecosystem [45]. In order to meet these requirements, physicians will need to have sufficient knowledge of mathematical concepts, the foundations of artificial intelligence, machine learning, data science, and related ethical and legal issues and be able to relate them in the context of *medicine* [46, 47]. Currently, medical education is facing a pedagogical problem pertaining to how and with what content AI should be introduced or included in the medical curricula, which is still in a controversial position in health services. Curriculums should not introduce AI to the students as a tool in an algorithmic way, instead should be based on the aspect that regulates and enriches students’ perceptions of clinical problems. Including principles of AI in the formal medical education will help the student to understand perceptual experiences in practice and complex clinical reasoning processes in medical applications with AI outputs used in medicine [2]. On the other hand, AI cannot yet offer a solution for direct communication, empathy and human touch, which have a very important place in healthcare. Based on these important differences, ethical debates on the relationship between the medical profession and artificial intelligence should definitely be included in the curriculum.

AI can be considered to be a key factor in the identity constructions of physicians of the future [2]. Since artificial intelligence continues to redesign the medical field, it will be imperative for physicians to know foundational artificial intelligence algorithms and techniques [44]. In order to attain the maximal efficiency from AI based technologies in medical practices and to protect the professional identity of the medical profession, curricular developments should be made in medical school programs to better understand the basic components and algorithms of artificial intelligence [47, 48]. For instance, it is stated that medical students who are trained in AI feel more secure in working with AI in the future than students who do not [14]. Another study suggests prior knowledge and readiness of AI will become a crucial skill for physicians in order to interpret medical literature, assess possible clinical software developments, formulating research questions [44].

This study developed the MAIRS-MS and evaluated its reliability and validity, which aimed to measure medical AI readiness of medical students. The overall results showed good reliability and validity of the MAIRS-MS in medical students. The scale consisted of 22 items, and EFA revealed that the MAIRS-MS had four factors: *cognition*, *ability*, *vision*, and *ethics* (Additional file 3). To investigate the concurrent criterion validity, the relationship of MAIRS-MS with a criterion (gold standard) measurement could not be applied as it is the first defined scale that is developed related to the subject.

The cognition factor of the readiness scale includes the items that measure the participant’s cognitive readiness in terms of terminological knowledge about medical artificial intelligence applications, the logic of artificial intelligence applications and data science. The ability factor of the scale includes items that measure the participant’s competencies in terms of choosing the appropriate medical artificial intelligence application, using it appropriately by combining it with the professional knowledge, and explaining it to the patient. The vision factor of the scale includes items that measure the participant’s ability to explain limitations, strengths and weaknesses related to medical artificial intelligence, anticipate opportunities and threats that may occur, and conduct ideas. Scale items under the ethics factor measure the participant’s adherence to legal and ethical norms and regulations, while using AI technologies in healthcare services.

Despite the rigor of this original first research, it suffers with some minor limitations. We collected the data from two public medical schools located within the same geographic region, and thus, the findings might not be generalized to most public and private medical schools. Additionally, the study was conducted only in Turkey; hence the results might not be generalizable in other countries, although the chances of this discrepancy are very minor. The convenience sampling approach applied in this study might cause possible selection bias. The findings presented in this study must also be carefully explored in the light of the differences across countries and cultures.

## Conclusions

To the best of our knowledge, the developed MAIRS-MS is the very first scale for assessing the perceived medical artificial intelligence readiness of medical students. Although this new scale is developed for medical students, we argue that it could also be used for measuring physicians’ medical AI readiness with needful modifications. However, due to lack of validity and reliability studies, the generalization of our findings for physicians and other healthcare professionals is restricted. Further, psychometric studies are warranted to investigate the replicating results of this study with physicians, residents, and other healthcare professionals. Studies in specific specialties (e.g., radiology) that pioneering the AI applications in healthcare would contribute the improvement of MAIRS-MS. In this way, a set of measurement tools can be produced that can assess the readiness in different healthcare fields for assisting future AI transformation.

The developed instrument MAIRS-MS through this study is innovative worldwide and it may contribute to the research on assessing medical students’ perceived artificial intelligence readiness. Also, the MAIRS-MS may provide benefit to medical schools, institutions, faculty members and instructional designers as a valuable curriculum development tool with its learner needs assessment opportunity. Besides, it could be beneficial in measuring the effectiveness of courses or trainings in AI-related curricula in medical schools. Another striking point is that the definition of medical artificial intelligence readiness is introduced first time here in this article.

## Supplementary Information

**Additional file 1:.** Five items that loaded on more than one factor and that were subsequently discarded.

**Additional file 2:.** Medical Artificial Intelligence Readiness Scale for Medical Students (MAIRS-MS). Confirmatory Factor Analysis Graphic

**Additional file 3:.** Medical Artificial Intelligence Readiness Scale for Medical Students (MAIRS-MS).

## Data Availability

The datasets used and/or analyzed during the current study are available from the corresponding author on reasonable request.

## Abbreviations

MAIRS-MS: Medical artificial intelligence readiness scale for medical students
AI: Artificial intelligence
EFA: Exploratory factor analysis
CFA: Confirmatory factor analysis
EU: Ege university
MCBU: Manisa celal bayar university
CI: Confidence interval
