# Supplementary material for: Medical artificial intelligence readiness scale for medical students (MAIRS-MS) – development, validity and reliability study
Source: BMC Med Educ. 2021 Feb 18;21:112. doi: 10.1186/s12909-021-02546-6 (PMC7890640; doi:10.1186/s12909-021-02546-6)
Supplement: Supplementary file 1 — Additional file 1:. Five items that loaded on more than one factor and that were subsequently discarded. [file 12909_2021_2546_MOESM1_ESM.docx]

**Additional file 1**

### **Five items that loaded on more than one factor and that were subsequently discarded.**

|  | 1 | 2 | 3 | 4 |
| --- | --- | --- | --- | --- |
| I can work as a team member with field experts in the process of developing AI applications. | 0.456 | 0.344 | -0.110 | 0.015 |
| I can follow the current developments and literature on the applications of AI in the field of health. | 0.469 | 0.177 | 0.060 | 0.440 |
| I can decide the usage of AI technologies in healthcare delivery. | 0.454 | 0.357 | 0.244 | 0.162 |
| I can keep health records in a way that AI applications can process. | 0.274 | 0.336 | 0.321 | 0.446 |
| I can explain how physician knowledge and experience are used in the development of AI applications. | 0.389 | 0.393 | 0.268 | 0.222 |
