# Supplementary material for: Medical artificial intelligence readiness scale for medical students (MAIRS-MS) – development, validity and reliability study
Source: BMC Med Educ. 2021 Feb 18;21:112. doi: 10.1186/s12909-021-02546-6 (PMC7890640; doi:10.1186/s12909-021-02546-6)
Supplement: Supplementary file 2 — Additional file 2:. Medical Artificial Intelligence Readiness Scale for Medical Students (MAIRS-MS). Confirmatory Factor Analysis Graphic [file 12909_2021_2546_MOESM2_ESM.docx]

**Additional file** **2**

**Medical Artificial Intelligence Readiness Scale for Medical Students (MAIRS-MS)**

**Confirmatory Factor Analysis Graphic**

**
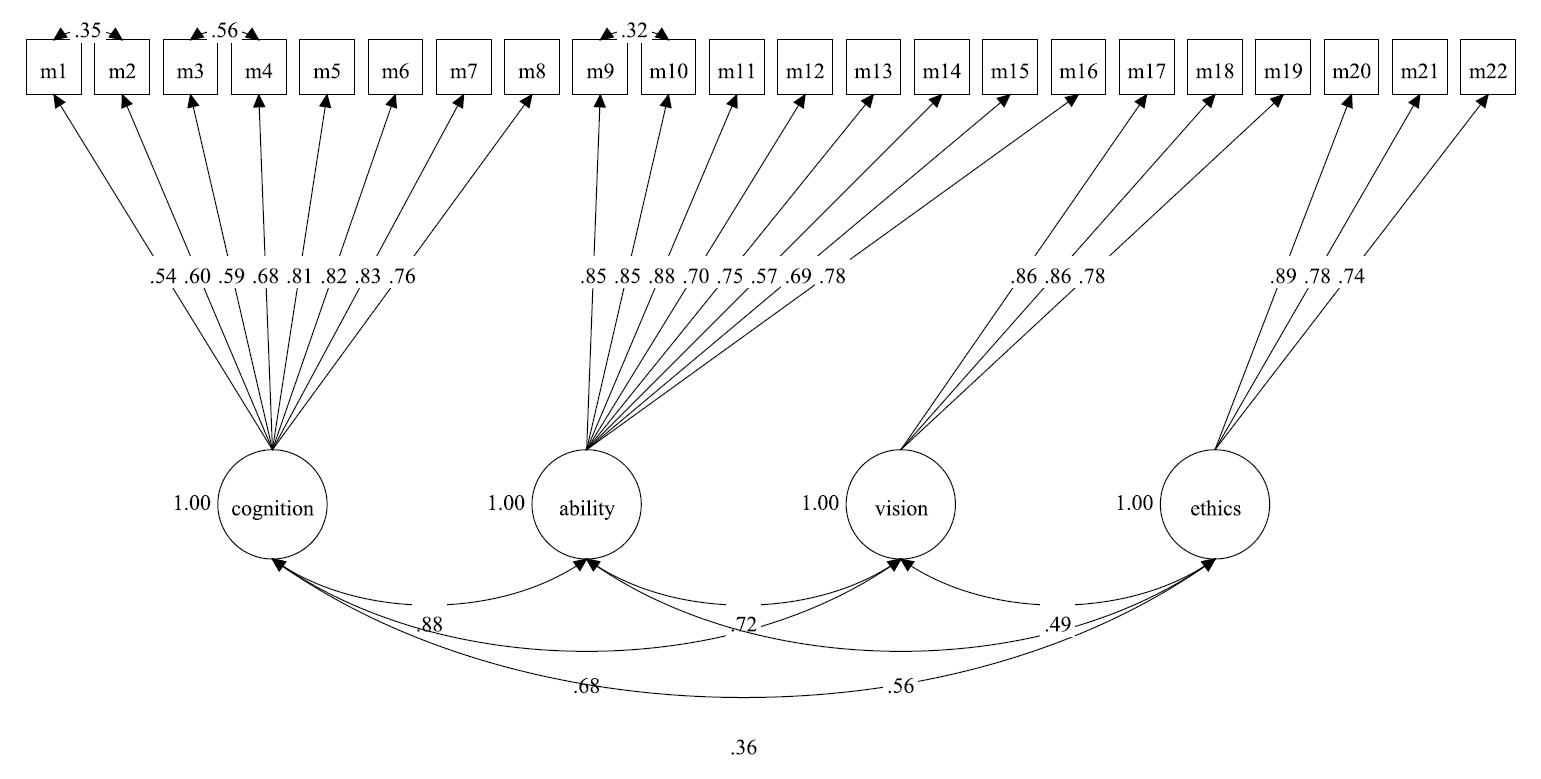
**
