# Supplementary material for: Medical artificial intelligence readiness scale for medical students (MAIRS-MS) – development, validity and reliability study
Source: BMC Med Educ. 2021 Feb 18;21:112. doi: 10.1186/s12909-021-02546-6 (PMC7890640; doi:10.1186/s12909-021-02546-6)
Supplement: Supplementary file 3 — Additional file 3:. Medical Artificial Intelligence Readiness Scale for Medical Students (MAIRS-MS). [file 12909_2021_2546_MOESM3_ESM.docx]

**Additional file 3**

### **Medical Artificial Intelligence Readiness Scale for Medical Students (MAIRS-MS)**

| 1-Strongly Disagree | 2-Disagree | 3-Neutral | 4-Agree | 5-Strongly Agree |
| --- | --- | --- | --- | --- |

| 1. I can define the basic concepts of data science |
| --- |
| 1. I can define the basic concepts of statistics |
| 1. I can explain how AI systems are trained |
| 1. I can define the basic concepts and terminology of AI |
| 1. I can properly analyze the data obtained by AI in healthcare. |
| 1. I can differentiate the functions and features of AI related tools and applications. |
| 1. I can organize workflows compatible with AI. |
| 1. I can express the importance of data collection, analysis, evaluation and safety; for the development of AI in healthcare. |
| 1. I can harness AI-based information combined with my professional knowledge. |
| 1. I can use AI technologies effectively and efficiently in healthcare delivery. |
| 1. I can use artificial intelligence applications in accordance with its purpose. |
| 1. I can access, evaluate, use, share and create new knowledge using information and communication technologies. |
| 1. I can explain how AI applications offer a solution to which problem in healthcare. |
| 1. I find valuable to use AI for education, service and research purposes. |
| 1. I can explain the AI applications used in healthcare services to the patient. |
| 1. I can choose proper AI application for the problem encountered in healthcare. |
| 1. I can explain the limitations of AI technology. |
| 1. I can explain the strengths and weaknesses of AI technology. |
| 1. I can foresee the opportunities and threats that AI technology can create. |
| 1. I can use health data in accordance with legal and ethical norms. |
| 1. I can conduct under ethical principles while using AI technologies. |
| 1. I can follow legal regulations regarding the use of AI technologies in healthcare. |

Cognition Factor: 1-8 Items Min:8 Max:40 points

Ability Factor: 9-16 Items Min:8 Max:40 points

Vision Factor: 17-19 Items Min:3 Max:15 points

Ethics Factor: 20-22 Items Min:3 Max:15 points

Medical Artificial Intelligence Readiness: 1-22 Items Min:22 Max:110 points
